# Orthrus: Towards Evolutionary and Functional RNA Foundation Models

**DOI:** 10.1101/2024.10.10.617658

**Published:** 2024-12-10

**Authors:** Philip Fradkin, Ruian Shi, Keren Isaev, Brendan J. Frey, Quaid Morris, Leo J. Lee, Bo Wang

**Affiliations:** 1 Vector Institute, Ontario, Canada.; 2 Computer Science, University of Toronto, Ontario, Canada.; 3 Computational and Systems Biology Program, Sloan Kettering Institute, New York, United States.; 4 New York Genome Center, New York, United States.; 5 Systems Biology, Columbia University, New York, United States.; 6 Electrical and Computer Engineering, University of Toronto, Ontario, Canada.; 7 Peter Munk Cardiac Center, University Health Network, Ontario, Canada.

## Abstract

In the face of rapidly accumulating genomic data, our ability to accurately predict key mature RNA properties that underlie transcript function and regulation remains limited. Pre-trained genomic foundation models offer an avenue to adapt learned RNA representations to biological prediction tasks. However, existing genomic foundation models are trained using strategies borrowed from textual or visual domains that do not leverage biological domain knowledge. Here, we introduce Orthrus, a Mamba-based mature RNA foundation model pre-trained using a novel self-supervised contrastive learning objective with biological augmentations. Orthrus is trained by maximizing embedding similarity between curated pairs of RNA transcripts, where pairs are formed from splice isoforms of 10 model organisms and transcripts from orthologous genes in 400+ mammalian species from the Zoonomia Project. This training objective results in a latent representation that clusters RNA sequences with functional and evolutionary similarities. We find that the generalized mature RNA isoform representations learned by Orthrus significantly outperform existing genomic foundation models on five mRNA property prediction tasks, and requires only a fraction of fine-tuning data to do so. Finally, we show that Orthrus is capable of capturing divergent biological function of individual transcript isoforms.

## 1 Main

Mature messenger RNAs (mRNA), resulting from transcription and splicing of precursor RNAs, encode essential genetic information for protein synthesis. The properties and functions of RNAs are often tightly linked to their sequence and are critical in modulating protein expression and cellular processes [1]. Experimental procedures [2–4] have been pivotal in studying RNA metabolism, but these techniques are often time-consuming and expensive. As an alternative, supervised machine learning models trained on genetic sequences can automatically extract RNA features from data, thus offering effective and low-cost prediction of post-transcriptional processes such as alternative splicing and RNA degradation [5–7]. These models can be used to identify disease mechanisms [8, 9], improve therapeutics such as mRNA vaccines [10], and predict the effects of perturbations [11]. Despite the importance of these applications, the difficulty associated with experimental acquisition of training data restricts the use of supervised methods for a wider range of tasks.

Several recent works [12–14] have proposed foundation models as an alternative for supervised learning approaches in genomic domains. Genomic foundation models use deep neural networks to learn an expressive representation of genetic sequences by pre-training on large datasets. During pre-training, self-supervised learning (SSL) objectives are used to train the model in the absence of labeled examples. SSL can be formulated through a data reconstruction objective, where a model is required to reconstruct a portion of the input data. Existing genomic foundation models use training objectives including next token prediction (NTP) and masked language modeling (MLM) [12, 15, 16]. Foundation models that effectively capture the underlying biological complexities enable few-shot learning, generalizing experimental biology using a minimal number of samples [17, 18]. Representations learned with foundation model techniques can be fine-tuned on related downstream tasks with fewer labeled data points, reducing reliance on data collection and demonstrating impressive generalization capabilities to a diversity of tasks [19, 20]. However, the unique properties inherent to genomic data pose challenges for implementing reconstruction-based SSL objectives or supervised learning approaches.

Genomic sequences in the natural world are constrained by evolutionary viability, resulting in low natural diversity^1^ and high mutual information across genomes from the same species [22]. Latest estimates propose that approximately ten percent of the human genome is under constraint and can be considered high information content [23, 24]. The remaining 90% of the genetic sequence lacks evidence of negative selection, meaning mutations may have little to no impact on organism fitness [25, 26]. Without a strong biological inductive bias, existing reconstruction-based SSL models often reconstruct non-informative tokens, which can result in sub-optimal representations. Due to the high-mutual information between samples, it is also difficult to scale the effective size of the training dataset to circumvent this issue. As we later show, applications of SSL methods to genomics learn latent representations that are not well suited for mRNA property prediction [12–14, 27]. In addition, current genomic foundation models are unable to incorporate exon co-ocurrence patterns, making it challenging to capture the distinct function and properties of individual RNA isoforms. The gap between baseline SSL methods and supervised approaches remains large, while no clear trend exists between model size and performance.

Here, we propose Orthrus, an RNA foundation model that is pre-trained on mature RNA sequences. Orthrus uses a novel biologically motivated contrastive learning objective to structure the model latent space by maximizing similarity between splicing isoforms and evolutionary related transcripts [28, 29]. Using this contrastive objective, Orthrus is pre-trained on splicing annotation data from 10 species and orthologous alignments from more than 400 mammalian species in Project Zoonomia [30]. Pre-training Orthrus on mature RNAs with high functional importance and sequence conservation further allows Orthrus to focus on sequence regions with high information content [23, 25]. Orthrus is trained using a Mamba encoder, which enables favorable model properties such as the learning of variable motif spacing, context filtration, and linear memory scaling with sequence length [31]. Orthrus pre-training results in effective mature RNA representations that are predictive of diverse mRNA properties and functions.

We show that Orthrus’s learned representations can be used to accurately predict the properties and functions of mature mammalian RNA sequences in three key contexts. First, we test the effectiveness of biologically inspired contrastive learning by fitting a linear model on top of the pre-trained latent representations. We identify that Orthrus outperforms other self-supervised foundation models, and applying this simple linear transformation approaches the performance of supervised methods on all property prediction tasks. Second, we fine-tune the pre-trained models on experimentally collected mRNA property datasets and demonstrate state-of-the-art performance when generalizing to unseen sequences. Orthrus is able to effectively perform in the low data regime, requiring as few as 45 labeled examples to fine-tune an mRNA half-life predictor. Finally, we demonstrate that Orthrus is capable of grouping RNA isoforms by their common functions in the model latent space. This brings us closer to being able to annotate the function of individual splice isoforms, a long-standing challenge in RNA biology.

The Orthrus model, including pre-trained weights and downstream evaluation datasets, is open-source and available at: https://github.com/bowang-lab/Orthrus.

## 2 Results

### Contrastive learning dataset construction

Orthrus is trained using contrastive learning, which constructs a structured representation space by directly maximizing the embedding similarity within positive pairs of related RNA transcripts while minimizing the similarity of all unrelated transcripts. Each positive contrastive pair consists of a reference RNA transcript that is paired with a transcript sampled from a set of related mature RNA sequences. Under the contrastive learning framework, these related mature RNAs can be viewed as augmentations of the reference transcript, as they share similarities in function and property but differ in sequence. We construct a contrastive learning dataset that identifies positive pairs based on alternative splicing and orthologous transcripts produced through mammalian speciation events. Our hypothesis is that positive pairs of mRNA sequences produced from these processes are more functionally similar to each other than randomly sampled RNA sequences.

**Fig. 1:**
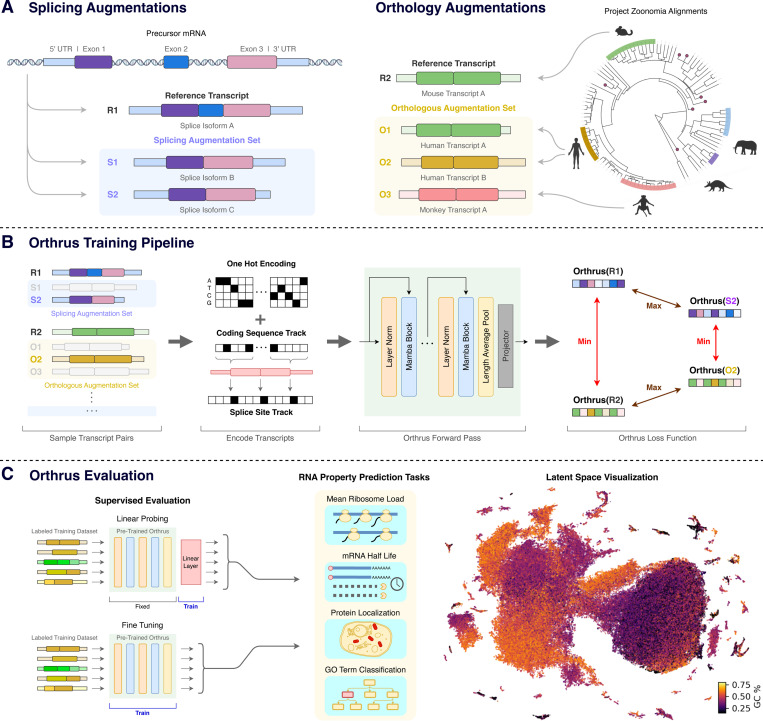
Overview of Orthrus. **(A) Contrastive dataset construction:** We treat each RNA transcript in the pre-training dataset as a reference transcript. For each reference transcript, we identify sets of transcripts that are related through alternative splicing and orthology. Each reference transcript can be associated to both splicing and orthology augmentation sets. **(B) Training pipeline:** For all reference transcripts in a batch, we randomly sample a positive paired transcript from its splicing and orthology augmentation sets. All transcripts are converted into a six track encoding. We then generate a projection of the sequences using Orthrus model and apply the contrastive loss over these samples, maximizing similarity between positive pairs while minimizing it for all the other transcripts. **(C) Evaluation:** Orthrus is evaluated in linear probing and fine-tuning contexts for a variety of mature mRNA property prediction tasks. Orthrus learns the structural properties of RNA transcripts, as shown by latent space visualization using UMAP [32].

To construct positive pairs based on alternative splicing, we group alternatively spliced transcripts using the GENCODE and RefSeq databases depending on availability [33, 34]. We use splice information across 10 metazoan organisms: human, mouse, chicken, C. elegans, chimpanzee, cow, dog, fruit fly, rat, and zebrafish (Figure 1 A). Alternatively spliced mRNA isoforms exhibit variability in UTR and coding sequences composition, at times demonstrating novel function. However, our work is based on the assumption that, on average, splice isoforms are more functionally similar to each other than randomly sampled mRNA transcripts. We empirically find that sequence diversity arising from different exon combinations in alternatively spliced isoforms serves as an effective source of function-preserving variation.

Orthologous transcripts from mammalian species present another source of function-preserving sequence diversity [35, 36]. We use positive pairs generated through the process of speciation across the Eutheria clade through the Zoonomia TOGA resource [30], which performs joint gene annotation and orthology inference mapping transcripts from over 400 species to human and mouse annotations. To identify orthologous pairs, TOGA performs alignment over identified coding sequences and neighboring intronic and intergenic regions. We hypothesize that orthologous transcripts have shared function, so we use them as positive pairs in our dataset, allowing the model to learn function-preserving sequence variation. Importantly, this exposes the model to transcript regions that are conserved over evolutionary time due to negative selection. These regions are likely to be functionally important and relevant for mRNA metabolism.

Overall, our final dataset contains 49 million unique transcripts and over 870 million unique positive pairs (Table 1, Section 4).

### Orthrus model overview

During the contrastive training phase, we sample positive pair sequences from mature RNA transcript sets and maximize their similarity in the model latent space (Figure 1 B). Given a batch of N reference sequences, $x_{1}^{1},\ldots ,x_{N}^{1}$, we construct positive pairs $\left(x_{i}^{1},x_{i}^{2}\right)$ by randomly sampling $x_{i}^{2}$ from the augmentation set of transcripts related to $x_{i}^{1}$ through alternative splicing or orthology processes, as described in the previous section. The augmentation set associated with each reference transcript can contain both splice isoforms and orthologous transcripts. The positive pair transcript from the augmentation set $\left(x_{i}^{2}\right)$ is re-sampled for each training epoch. We pass these positive pairs through a Mamba [31] encoder $\left(f_{\theta }\right)$ resulting in the outputs $h_{i}^{1}$ and $h_{i}^{2}$. These representations are then fed into a multi-layer perceptron projection head $\left(g_{\theta }\right)$ and the outputs are used to calculate normalized projections $z_{i}$. We use the decoupled contrastive learning (DCL) loss [37] to perform the contrastive learning objective, pushing apart unpaired transcripts and maximizing the cosine similarity between positive pairs (Figure 1 B). After pre-training, we discard the projection head and directly use the outputs from the Mamba encoder as embeddings. We introduce two versions of Orthrus using a backbone Mamba encoder: Base consisting of 1.3 million trainable parameters and Large with 10.1 million trainable parameters (excluding $g_{\theta }$). Model hyperparameters are reported in Appendix A.1.

### Orthrus embeddings are predictive of diverse mRNA properties

To evaluate the effectiveness of our pre-trained representations, we followed the conventional evaluation strategy of linear probing. The learned latent embedding is effective if ∃w s.t. wTX+b=y^, where, X is a matrix of embeddings, and y^ are the predictions for labels y. To evaluate the above, we freeze the weights of the Mamba encoder $f_{\theta }$ and train a linear layer to predict labels for regression and classification tasks. Further experimental details are described in Appendix A.3.

**Fig. 2:**
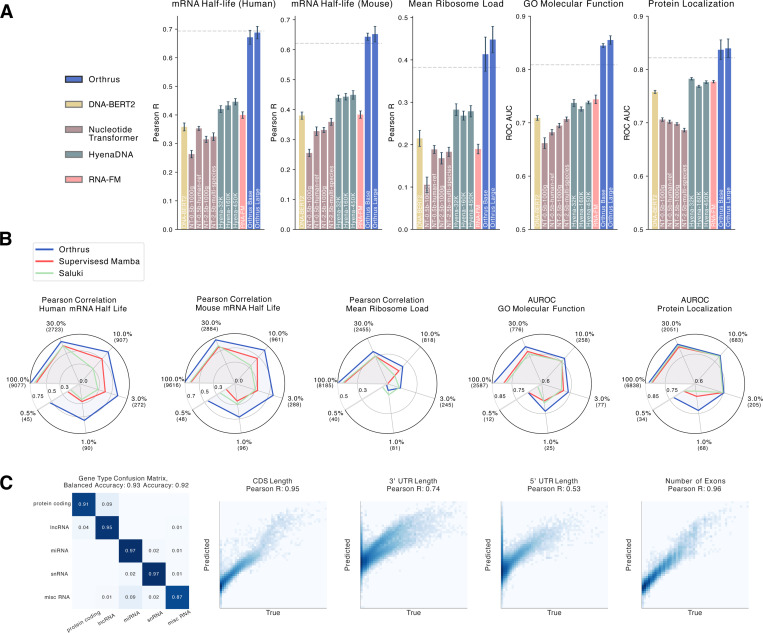
mRNA Property Prediction using Orthrus. **(A)** Benchmarking linear probing performance on mRNA property prediction tasks for self-supervised genomic foundation models. Individual bars represent the performance of foundation model variants, which typically differ in parameter count and pre-training dataset. Error bars show 95% confidence intervals, constructed using 10 runs with randomized data splits. The grey dashed line indicates the performance of the fully fine-tuned supervised Saluki method. **(B)** Plots evaluating the fine-tuning performance of Orthrus Base across varying levels of data availability. Each dataset is subsampled to the indicated percentage, with the number of data points provided in brackets. Point estimates are plotted, averaged across three random seeds and random data splits. **(C)** Evaluation of Orthrus’s latent representation by fitting a linear model to predict structural properties. The confusion matrix evaluates Orthrus’s ability to classify transcript types using logistic regression on learned embeddings. The four scatter plots assess Orthrus’s ability to predict structural RNA properties, including CDS length, 3’ UTR length, 5’ UTR length, and number of exons.

We quantitatively evaluate whether Orthrus embeddings contain information regarding key transcript properties such as UTR length, number of exons, CDS length, and transcript type in Figure 2 C. We observe that Orthrus embeddings are highly predictive of these attributes, which are important for predicting properties such as mRNA half-life [7]. We note that Orthrus embeddings are of a fixed length, meaning these properties cannot be simply derived as a function of embedding dimensionality. In Figure 2 A, we demonstrate that Orthrus outperforms other self-supervised methods on a diverse set of mRNA property prediction tasks by a substantial margin. For mRNA half-life (Human), Orthrus Large outperforms other self-supervised methods, with the closest competing model achieving only 65% of Orthrus’s linear probing accuracy (Pearson’s R 0.69 vs 0.45, respectively). Furthermore, we evaluate a Base 4-track model and find that Orthrus still outperforms other self-supervised baselines using only sequence information (Table A2). For each task, we also train an *ab-initio* supervised deep learning model with full access to the ground truth labels. We use the CNN-RNN hybrid architecture from Saluki [7] for this supervised model, as it has been shown to offer state-of-the-art performance on mRNA property prediction. The results for each model are shown with a dashed line in Figure 2 A. For all mRNA property prediction tasks, we find that Orthrus embeddings match or outperform the supervised baseline. These results indicate that a simple linear regression trained on Orthrus embeddings can replace the expensive task-specific training of neural networks for mRNA property prediction.

We observe improved linear probing results as we scale the number of trainable parameters for Orthrus by comparing Base and Large model variants (Figure 2). We find that this trend of improvement is strongest in MRL and GO Molecular Function predictions. We note that for other self-supervised models such as Hyena DNA [14] or Nucleotide Transformer [27], increasing the number of parameters does not consistently improve performance (Figure 2 A). However, we do observe an improvement in performance for Nucleotide Transformer when comparing their 2.5 billion parameter model pre-trained on 1000 genomes data versus multi-species. This is additional evidence that using evolutionary information can help improve model performance on mRNA property prediction tasks [38].

### Fine-tuning Orthrus for state-of-the-art mRNA property prediction

**Fig. A2:**
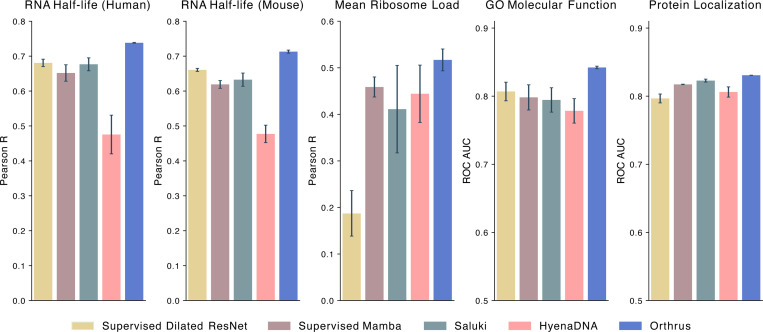
Fine tuning results plotted for 100% fraction of the data. Standard deviation is computed across 3 random seeds. Datasets are split with a homology aware strategy to avoid data leakage.

To assess whether the Orthrus pre-training objective provides utility beyond an effective representation, we evaluate its performance by fully fine-tuning it and comparing it to a supervised model with a matched architecture. We compare its performance against a published method for the mRNA half-life prediction, Saluki [7], and find that the fully fine-tuned Orthrus model outperforms Saluki on the mRNA half-life task (Figure 2). Furthermore, we retrain the Saluki architecture, train an architecturally equivalent model to Orthrus, and fine-tune HyenaDNA for the other sequence property prediction tasks and identify that Orthrus has a significant performance advantage (Figures 2, A2). Other baseline SSL methods such as DNA-BERT2 and RNA-FM have limited input context windows, and cannot be easily applied to these tasks.

To simulate downstream tasks for which there is a lack of experimental data, we perform fine-tuning on mRNA property prediction tasks where only a subset of the original training data set is available. We observe that supervised methods are ineffective in this regime, while Orthrus maintains competitive performance at 10% and 1% of the data (Figure 2 B). The performance differences are even more stark when using only 0.5% of the training data, achieving 73% of supervised performance with just 45 observed samples on the human mRNA half-life dataset (Pearson’s R = 0.72 vs Pearson’s R = 0.53). These findings illustrate that Orthrus advances towards the aim of few-shot learning for downstream tasks where experimental data is scarce.

### Orthrus latent space encodes functional similarities

**Fig. 3:**
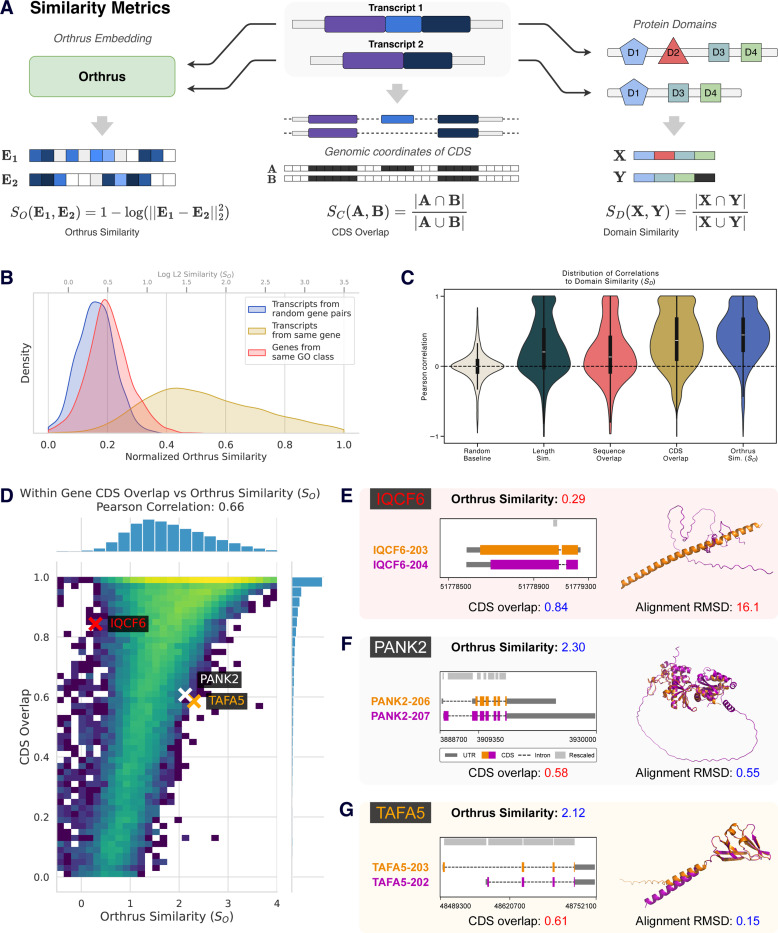
Analysis of functional similarities in Orthrus embeddings. **(A)** Methodology for comparing gene isoform similarities using Orthrus embeddings, protein domain annotations, and CDS overlap. Orthrus embeddings for transcripts within the same gene are compared using log of L2 distance, while protein domain similarity and CDS overlap is computed using the Jaccard index. **(B)** We visualize within gene similarities (yellow), between gene similarities (blue), and similarities of genes from the same GO class (red). **(C)** Visualization of Pearson’s R distributions correlating protein domain similarities with baseline or Orthrus embedding similarities for 1000 randomly sampled genes with multiple isoforms. **(D)** Heatmap illustrating the relationship between CDS overlap and Orthrus similarity in pairs of alternatively spliced transcripts. **(E-G)** Comparative analysis of transcript pairs where the CDS overlap and Orthrus similarity metrics are divergent. AlphaFold3-predicted structures for isoforms are superimposed. Orthrus similarity captures structural similarity as quantified by root-mean-squared deviation (RMSD). Genomic coordinate plots for individual isoforms are also shown.

Having shown that Orthrus captures properties of individual transcripts, we then investigated its ability to detect variation in function among different isoforms of the same gene [39]. To explore this, we compute Orthrus embedding similarities for each pair of protein-coding transcripts of the same gene (Figure 3 A). As a control, we compare these with the transcript pairs of random genes, expecting a lower similarity. We also hypothesized that transcript pairs from genes sharing the same GO terms would be more similar than random pairs, but less similar than most intragene pairs. Our analysis confirms significant differences across all pairwise comparisons of the three groups (p < 2.2e-16, Mann-Whitney U test), indicating that the Orthrus training objective preserves within gene sequence diversity (Figure 3 B). Notably, we observe an overlap between intragene and intergene similarities, indicating that some alternatively spliced transcripts have distinct embeddings, RNA properties, or functional differences in protein products. As such, *within gene* diversity could potentially help delineate differential isoform protein functions, an active area of research.

To investigate whether Orthrus similarities for intragene transcripts reflect functional similarity, we compared annotated protein domains across each transcript pair (Figure 3 A). We find that transcripts with a high degree of protein domain overlap ($S_{D}$) also have highly similar Orthrus embeddings ($S_{O}$) with a median Spearman’s rho and Pearson’s R correlations of 0.37 and 0.45 respectively. This correlation is significantly higher than when compared to baseline metrics such as transcript length and overall sequence overlap, indicating that Orthrus better captures functional differences encoded by protein domains (Figure 3 C). We expect that transcript sequence overlap, especially in coding regions, is likely to contribute to similarities in RNA embeddings, and visualize the relationship between CDS overlap (see Methods) and Orthrus similarity for intragene pairs of protein-coding isoforms across all genes (Figure 3 D). An overall positive correlation is observed (Spearman’s rho = 0.68, Pearson’s R = 0.66), confirming that Orthrus identifies the coding sequence as a major determinant of function. However, we observe that Orthrus captures variability in functional similarity that extends beyond sequence similarity.

In the panels shown in Figure 3 (E-G), we investigate individual examples where transcript pairs have high CDS overlap but low Orthrus embedding similarity (*IQCF6*) and the reverse, where sequences share a smaller fraction of the sequence and yet their embeddings are close, as indicated by Orthrus similarity (*PANK2, TAFA5*). Isoforms in *IQCF6* have a very similar coding sequence (0.93) but a low Orthrus similarity (0.84, 1.52^*th*^ percentile), suggesting our model predicts them to be functionally distinct. To evaluate the functional differences between these splice isoforms, we generate structure predictions of the transcripts’ coding sequences with AlphaFold3 [40] and structurally align them using PyMOL [41]. The structures demonstrate low overall alignment, as evidenced by a Root Mean Square Deviation (RMSD) of 16.1 angstroms (Å), inline with our Orthrus-based scoring. Meanwhile, isoforms in both *TAFA5* and *PANK2* exhibited low coding sequence overlap (Jaccard Index < 0.6, bottom 20^*th*^ percentile). However, Orthrus embedding similarity is predicted to be high (60^*th*^ and 55^*th*^ percentiles respectively). Similarly, we visualize the predicted structures for the pairs of transcript coding sequences and find low overall RMSD values of 0.15Å and 0.55Å, confirming structural similarity despite low coding sequence overlap. Cumulatively, these findings suggest that Orthrus embeddings encode functionally relevant information.

### Orthrus Embeddings Capture Functional Transcript Diversity

**Fig. 4:**
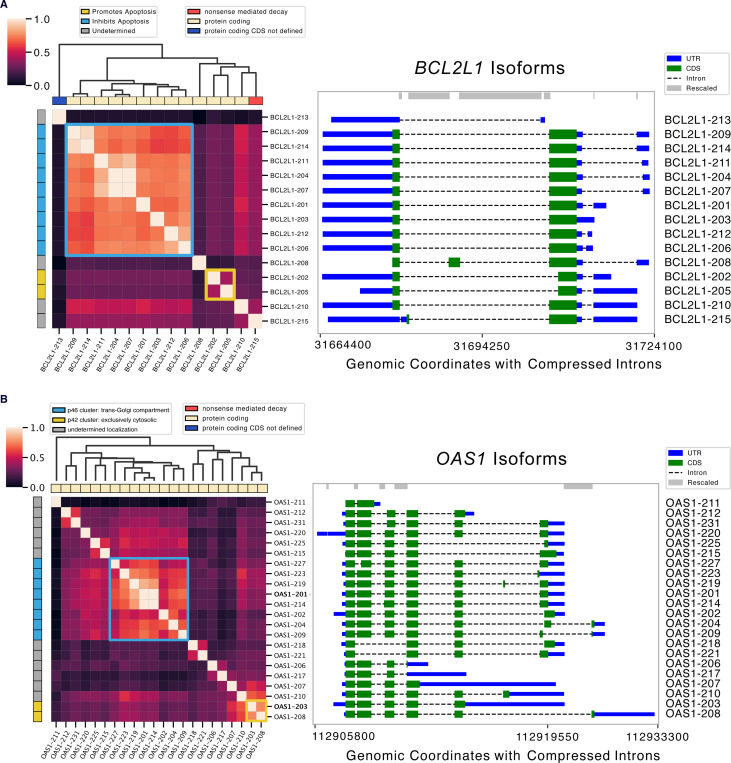
Functional clustering of splice isoforms using Orthrus. Heatmaps visualize normalized Orthrus embedding similarity between all splice isoforms within the analyzed gene. Transcript biotype is annotated along the x-axis, while functional annotations for isoforms are displayed along the y-axis. Isoform genomic coordinate plots are shown on right. The clustering matrix, derived from Orthrus embedding similarities, is represented by the dendrogram. Highlighted cells in the heatmap indicate clusters with divergent transcript functions. **(A)** *BCL2L1* isoforms where apoptosis-inhibiting isoforms cluster together, while non-coding and apoptosis-inducing isoforms display low similarity. **(B)** *OAS1* isoforms clusters represent distinct cellular localization trends associated with viral response.

We investigate whether Orthrus is able to capture well-studied examples of intragene divergent function by clustering learned embedding similarities. To illustrate this, we select two genes that are experimentally annotated with distinct isoform function and localization. We first examine *BCL2L1*, known for its alternatively spliced isoforms with distinct roles in the apoptosis pathway [42, 43]. The dominant isoforms encode an apoptosis-inhibiting protein, Bcl-X(L), while a minority encode a pro-apoptotic protein, Bcl-X(S). By clustering *BCL2L1* RNA isoforms using Orthrus embedding similarity, we identify two main functional groups: one containing *BCL2L1–202* and *BCL2L1–205*, distinct from the apoptosis-inhibiting transcripts cluster (Figure 4 A).

As another example, the two major isoforms of *OAS1*, p42 and p46, have previously been shown to have distinct antiviral functions [44]. Prenylation, a post-translational modification that adds a hydrophobic lipid group to a protein, allows the p46 (*BCL2L1–201*) isoform to anchor to membranous organelles such as the trans-Golgi compartment and block the replication of viruses such as SARS-CoV. However, the p42 (*BCL2L1–203*) isoform lacks prenylation and does not inhibit viral replication, highlighting functional divergence between isoforms [44]. Using Orthrus embedding similarities, we show that these two distinct isoforms form their own marked clusters (Figure 4 B). This demonstrates that Orthrus embeddings may serve as a valuable resource for identifying isoforms with likely different functional properties, a critical area in alternative splicing research.

## 3 Discussion

In this work, we introduce Orthrus, a mature RNA foundation model that is trained to capture the diversity of RNA through an evolutionary and functional lens [45, 46]. We create a self-supervised training objective that learns similarities between evolutionarily related sequences identified in the Zoonomia project [30]. In addition, we utilize alternatively spliced transcripts to learn sequences responsible for shared functions between splicing isoforms [47]. By training on sequences generated by evolutionary and alternative splicing processes, Orthrus utilizes stronger biologically motivated inductive biases compared to SSL reconstruction methods. This makes Orthrus less reliant on limited genetic sequence diversity during pre-training, and capable of learning strong representations without fine-tuning on experimental data.

Previous self-supervised works for genomic sequence property prediction have focused on reconstruction objectives like masked language modeling or next token prediction [12, 27]. However, most positions in the human genome are under little to no negative selection, and are not as informative for model training [23, 24]. Thus, predicting the corresponding tokens introduces little new information to the model.

We demonstrate that by minimizing the distance between mature RNAs generated through speciation and alternative splicing, Orthrus can generate representations useful for mRNA property prediction tasks. We empirically demonstrate that Orthrus embeddings contain information useful for predicting properties like mRNA half-life and mean ribosome load, and achieves state-of-the-art prediction when fine-tuned. We observe that pre-training is especially helpful in low data regimes when there are 200 or fewer data points with labels. We demonstrate that carefully designed self-supervised pre-training mitigates the data efficiency challenges present in genomics, and that scaling to additional species can be an effective dataset expansion strategy.

An important question to address is why we would expect distance minimization between RNA isoforms to be useful for predicting properties like mRNA half-life or protein localization. One hypothesis is that alternative splicing and speciation events preserve core functional RNA segments. Through the contrastive pre-training procedure, we identify these shared regions between diverse sequences. Indeed, a recent work proposes that contrastive methods are effective due to block separating latent variables shared between augmented samples [48]. This view is supported by the observation that Orthrus intragene similarities are correlated with domain presence. By using decoupled contrastive learning, diverse sequences are pushed apart, thus uniformly distributing samples in the latent space, which helps with downstream task performance [37, 49]. Through encoding these invariances, we find that Orthrus is able to learn complex mRNA properties such as mean ribosome load and mRNA half-life.

A possible limitation of our approach is that by maximizing similarity in representation space between functionally related sequences, we remove important signals for predicting properties. Are there property prediction tasks for which our inductive bias is actually detrimental compared to a randomly initialized model? For mRNA half-life, [50] demonstrated that in more than 85% of genes, isoform choice has no statistically discernible effect. We find that intragene Orthrus embeddings still demonstrate significant diversity, demonstrating capability of reflecting intragene variable function. There are other processes for which it is widely considered that alternative splicing is functionally crucial, such as the development of neurological tissues [51, 52].

Despite the Orthrus training objective, which clusters alternatively spliced isoforms in the model’s latent space, our results show that Orthrus effectively captures transcript-specific functions. As illustrated in Figure 3, Orthrus representation similarities reveal functional divergence among isoforms, even when they share extensive sequence overlap. Further, Figure 4 demonstrates that Orthrus can group alternatively spliced transcripts based on their distinct functional roles. This capacity to discern transcript-level functionality is especially valuable given the scarcity of transcript-level functional annotations, as it enables more detailed biological insights. Conditioning these analyses on properties such as protein localization or mRNA stability, for instance, could guide investigations into even more nuanced aspects of transcript-level functional variation.

In this work, we propose a novel, self-supervised contrastive objective for learning mature RNA isoform representations. We show that this approach is an effective strategy to address two major challenges for cellular property prediction: data efficiency, and model generalizability. We demonstrate that Orthrus representations are effective in the low data setting, paving the path to true few-shot learning for mRNA property prediction. Finally, we outperform supervised models when fine-tuning Orthrus and significantly improving over performance of reconstruction based self-supervised methods. These findings open the possibility that combining the contrastive loss with a masked language modelling objective can further improve quality of mature RNA representations.

## 4 Methods

Contrastive learning has been shown to be a bound on mutual information between two random variables X and Y corresponding to $I(X;Y)=E_{p(x,y)}\left[log\frac{p(x,y)}{p(x)p(y)}\right]$. We utilize a variation of the classical InfoNCE loss, $E\;\left[log\frac{\exp\left(f\left(x_{i},y_{i}\right)\right)}{\Sigma \exp\left(f\left(x_{i},y_{j}\right)\right)}\right]$, where a model f is tasked with classifying the correct $y_{i}$ which was jointly drawn with $x_{i}$ [53]. Herein, the observations $x_{i},y_{i}$ correspond to splice isoforms or orthologous sequences which are interpreted as functionally related while f is a neural network that we optimize to minimize the loss.

We propose to use four different augmentations and thoroughly investigate their impact on downstream tasks. They include: alternatively spliced transcripts across ten organisms, orthologous transcripts identified from the Zoonomia project including over 400 species, naive orthology informed by gene identity, and masking 15% of the input sequence (Figure 1) [30, 34].

In the following section we elaborate on dataset construction, model choice, contrastive learning objective, and downstream evaluations.

### Splicing and Orthology Contrastive Dataset

In the computer vision domain, contrastive learning strategies have had significant success by identifying augmentations that do not have a strong semantic effect, such as cropping, rotation, or Gaussian blur [29, 54, 55]. In this work, we use RNA splicing isoforms and orthologous transcripts as sources of functional similarity [30, 33, 34]. By sampling RNA isoform sequences produced by alternative splicing and speciation processes, we identify sequence variation that is likely to maintain core functional properties. In addition, we use naive orthology to pool RNA transcripts from evolutionarily related genes [47]. Here, for cases where gene names are consistent between species, we pool the transcripts generated by alternative splicing into the same transcript set. By minimizing the distance between functionally similar sequences, the model can learn regulatory regions critical for mRNA property and function prediction.

We generate a six-track mature RNA representation, consisting of four one-hot encoded tracks encoding genomic sequence, a track indicating the 5’ location of splice sites, and a track indicating the first nucleotide of every codon in the CDS. The addition of splice site and coding sequence locations has been shown to be beneficial for mRNA property prediction tasks [7].

To sample positive pairs from the orthology and splicing dataset, we first identify the set of all positive samples $Y_{j}$ for a reference transcript $x_{j}$. $Y_{j}$ can be variable in length since some transcripts will have a greater number of splice isoforms and orthologous sequences than others. During a forward model pass, we sample $y_{j}^{k}$ from $Y_{j}$ and use that as a positive pair for $x_{j}$.

### Mamba Encoder

We pre-train a Mamba state space model, which has been demonstrated to be successful in applications with long context requirements [14, 31]. mRNA sequences can reach over 12,000 nucleotides in length, making application of the Transformer architecture challenging due to its quadratic scaling in memory with sequence length [56]. Mamba, an extension of state space model families and S 4 [57], maps a sequence x(t)∈R to y(t)∈R using a latent state $h(t)\in R^{N}$.

A fundamental trade-off in architecture choice for sequence modeling is avoiding compressing sequence context and compute requirements. Transformers are able to avoid compressing context, leading to better performance, but trade-off slower training and higher memory usage [31, 56]. Alternatively, S4 models define a sequence to sequence transformation parameterized by (A, B, C, Δ). The fundamental operation consists of iteratively updating the hidden state:

$$\begin{array}{r} h^{\prime }(t)=A\;h(t)+B\;x(t)\\ y(t)=C\;h(t). \end{array}$$

Δ is used to discretize the input for discrete domains such as natural language, or genomics. The Mamba architecture iterates on the S4 family of models by introducing selectivity over input by making B, C, and Δ a function of the input, resulting in

$$\begin{array}{r} h^{\prime }(t)=A\;h_{t}+B\;\left(x_{t}\right)x_{t}\\ y(t)=C\;\left(x_{t}\right)h_{t}. \end{array}$$

Allowing parameters to be input dependent introduces desirable modeling qualities for genomic domain: variable spacing, filtering context, and linear memory scaling with sequence length O(n). Variable spacing refers to Mamba’s ability to effectively perform on the selective copying task, where causal elements are arbitrarily spaced [31]. Binding motifs in genomic sequences can be spaced without a constant offset, requiring the model to be able to learn motif interactions with variable spacing [58]. The non-unformity of signal informativeness in genomic sequences requires models to be able to filter out irrelevant context [31]. Finally, the limited context, as opposed to Transformer models, allows the Mamba architecture to scale required memory linearly with increased input length [31, 56].

### DCL Contrastive Learning Objective

We use decoupled contrastive learning (DCL) as it has been shown to require smaller batch sizes, is less sensitive to hyperparameters such as learning rate, and the positive loss term can be weighted by sample difficulty [37]. DCL iterates on the normalized temperature-scaled cross-entropy loss by splitting the contrastive objective into two terms: a similarity loss (positive) and a dissimilarity loss (negative) [59]. More formally, the positive and negative losses for sample *i* are calculated:

(1) $${𝓛}_{DCL,i}(\theta )=log\left[\sum_{k=1}^{N}\;\sum_{l=1}^{2}{𝟙}_{k\neq i}\exp\left(\langle z_{i}^{1},z_{k}^{l}\rangle /\tau \right)\right]-w_{i}\langle z_{i}^{1},z_{i}^{2}\rangle /\tau .$$

In the above $z^{1}$ and $z^{2}$ correspond to two embeddings of related sequences, $z_{k}$ are embeddings from unrelated RNA sequences, τ is the temperature parameter set to 0.1, and ${𝟙}_{k\neq i}$ is an indicator function that evaluates to 1 when k≠i. The above loss is computed for all the samples in the batch for both the sampled views l∈1, 2. N corresponds to all the negative samples in batch, thus maximizing batch size during contrastive learning typically leads to improved performance.

Normalized projections $z_{i}$ are outputs from the MLP projector $g_{\theta }$ and are used to compute the contrastive loss, utilizing samples from the rest of the batch as negative examples:

(2) $$z_{i}^{1}=\frac{g\left(h_{i}^{1}\right)}{\left\|g\left(h_{i}^{1}\right)\right\|}\;\text{and}\;z_{i}^{2}=\frac{g\left(h_{i}^{2}\right)}{\left\|g\left(h_{i}^{2}\right)\right\|}.$$

For downstream RNA property evaluations, the projector $g_{\theta }$ is discarded and outputs from $f_{\theta }$ are used instead. This practice is consistent with prior literature [29, 60–62].

### Downstream Evaluation Tasks

**mRNA half-life (mRNA HL)** is an important cellular property to measure due to its implications for protein expression regulation. Recently, it has been shown that the choice of experimental methodology for measuring mRNA half-life can have an outsized impact [7]. To address this challenge, Agarwal and Kelley (2022) utilized the first principal component of over 40 different mRNA half-life experiments. The dataset consists of 10,432 human and 11,008 mouse mRNA sequences with corresponding measurements. The low data availability and high inter-experiment variation underscore the importance of data efficiency, and generalizability in computational models to be developed for this task.

**Mean ribosome load (MRL)** is a measure of the translational efficiency of a given mRNA molecule. It measures the number of ribosomes translating a single mRNA molecule at a point in time. Accurate MRL measurement is crucial as it offers insights into the efficiency of protein translation, a key process in cellular function. The dataset in question, derived from the HP5 workflow, captures this metric across 12,459 mRNA isoforms from 7,815 genes [63]. This dataset was derived from a single experiment, so we can expect a higher amount of noise associated than the mRNA half-life dataset.

**Protein localization** describes a protein’s subcellular location, which can be determined using cells that are immunofluorescently stained. Protein function is often linked with its localization, underscoring the importance of this task. We downloaded a dataset of 10,409 genes, whose protein localization was determined by the Human Protein Atlas [64]. We included the 12 most common locations including Nucleoplasm, Cytosol, Vesicles, Mitochondria, Plasma Membrane, Golgi apparatus and others. We utilized one transcript per gene (defined to be the canonical isoform by the Appris database [65]).

**Gene ontology (GO)** terms are a hierarchical classification system used for assigning function to genes and their products [66–68]. In this work, we utilize GO classes to visualize model latent embeddings and classification. GO term hierarchical systems allow for fine-grained annotation of function, with broader terms at the top of the hierarchy and increased specificity closer to the bottom. To annotate genes with gene ontology terms, we subset GO classes three levels from the root, labeling all available genes.

### Associating Orthrus RNA Embeddings with Transcript Similarity and Protein Domains

To evaluate how well Orthrus RNA embeddings capture functional diversity among transcript isoforms, we analyzed the similarity of transcript pairs within and between protein-coding genes, excluding homologous genes when comparing random gene pairs or genes sharing the same GO term. The test dataset for this analysis was prepared as follows:

1. **Intragene Pairs**: We sampled 1,000 genes to obtain pairs of protein-coding transcripts.
2. **Intergene Pairs**: We randomly sampled 1,000 pairs of non-homologous genes, selecting the MANE transcript for each gene, which represents the most likely relevant isoform.
3. **Inter-gene Pairs**: We sampled 5,000 GO terms, each containing 10 to 1,000 genes, and selected five non-homologous gene pairs per term.

For each transcript, we computed Orthrus embeddings and calculated pairwise distances between embeddings using the L2 norm. We calculated a similarity score for each transcript pair as 1 − log(L2 distance). This ensures more interpretable results, where higher similarity scores correspond to closer RNA embeddings in the latent space, allowing us to compare the three groups of transcript pairs.

To assess whether similarities in Orthrus embedding reflected shared functional features, we annotated each transcript with protein domain information using Ensembl data and the Pybiomart package. We used the Jaccard Index to quantify the similarity of protein domain presence or absence between each pair of transcripts within a gene. The Jaccard Index is defined as the size of the intersection divided by the size of the union of the protein domain sets present in each transcript pair:

$$\text{Jaccard Index}=\frac{\left|D_{1}\cap D_{2}\right|}{\left|D_{1}\cup D_{2}\right|}$$

where $D_{1}$ and $D_{2}$ are the sets of protein domains present in each transcript. Higher values indicate greater similarity in protein domain composition. We calculated this metric using “intragene pairs” and “intergene pairs” to further study how protein domain composition correlated with embedding similarity. We analyzed the Pearson correlation between Jaccard indices and embedding similarities separately for intragene and intergene pairs to determine if transcript pairs within the same gene exhibited higher concordance. To calculate sequence overlap, we take either the full set of transcript genomic intervals (sequence overlap) or just the coding sequence (CDS overlap) and calculate similarity using Jaccard index as indicated above. This way, two fully overlapping sequences will have a Jaccard index of 1 and non-overlapping will be 0.

To generate structural alignments we use the AlphaFold3 server to fold protein sequences as identified in Ensembl v113 [69, 70]. We then take the 0^*th*^ seed from generated structures and generate an alignment using PyMOL [41].

To further explore the utility of Orthrus embeddings, we conducted a detailed analysis of the *BCL2L1* and *OAS1* genes [42, 44]. Transcripts from this gene were clustered based on their Orthrus embedding similarity scores, with clusters visualized and annotated according to transcript type and known functional roles.

## Figures and Tables

**Table 1:** Overview of contrastive datasets.

Contrastive Dataset	Zoonomia	Splicing	# of Pairs	# of Transcripts
Zoonomia Eutheria & Splicing Gencode Basic	**✓**	**✓**	876,871,640	49,493,993
Zoonomia Eutheria	**✓**	**✗**	157,975,815	41,562,358
Splicing Gencode Basic	**✗**	**✓**	16,249,112	771,105
None	**✗**	**✗**	0	771,105

**Table 2:** Overview of evaluation datasets.

Task Dataset	Category	Number of Sequences	Maximum Sequence Length	Homology Split Possible	Species
RNA Half Life Human	Regression	12968	12288	**✓**	Human
RNA Half Life Mouse	Regression	13738	12288	**✓**	Mouse
Mean Ribosome Load	Regression	11693	12275	**✓**	Human
Protein Localization	Classification	9769	12275	**✓**	Human
Gene Ontology MF	Classification	3697	12236	**✓**	Human

**Fig. A1:**
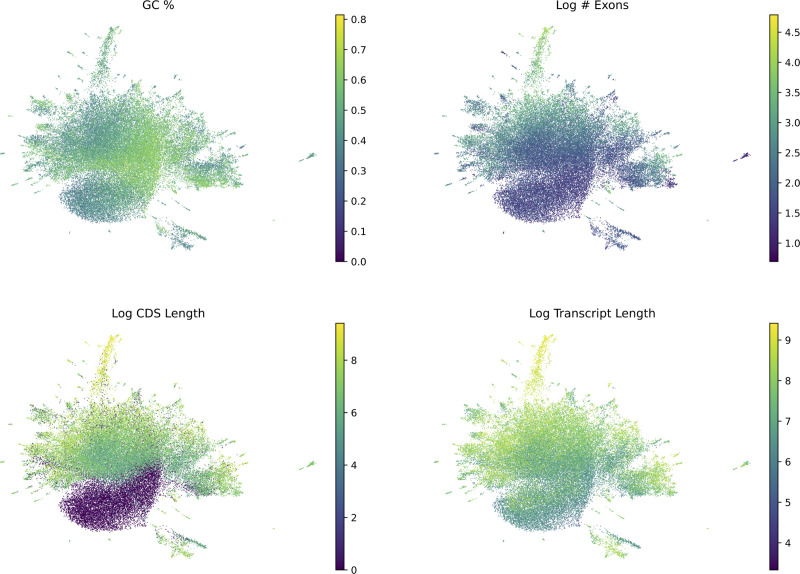
UMAP visualization of the Orthrus Large embeddings. 50,000 transcripts are randomly sampled from gencode comprehensive and colored according to transcript length, number of exons, CDS length, and GC%.

## Footnotes

1 In the coding region (2% of human DNA), an average individual carries 27 ± 13 unique SNPs [21].

## Appendix A

### A.1 Model Architecture and Pre-training Hyperparameters

Here, we report the model architecture and hyperparameters used to train the Orthrus model. The Orthrus Base model uses a 3-layer Mamba block with a hidden dimension of 256, while the Orthrus Large model uses a 6-layer Mamba block with a hidden dimension of 512. All other hyperparameters are kept to their default value. Due to computational constraints, larger models were not evaluated. Orthrus uses a two layer projection head with a hidden layer size of 512, and projection dimension of 128. The network uses ReLU activations and BatchNorm between layers.

Orthrus was trained using either four A100 or H100 GPUs using PyTorch’s DDP. Ablation experiments were performed using four A40s. Due to the skewed distribution of transcript lengths, batches were constructed to minimize sequence padding. During each training iteration (corresponding to four batches, each of which is sent to an individual GPU), we create one smaller batch to contain longer transcripts, while the remaining three batches are larger with short transcripts. We found that using a sequence length threshold of four thousand maximized GPU utilization, with resulting batch sizes of 50 and 150 respectively. Exact batch sizes were adjusted to maximize memory utilization on the GPU type used for each pre-training run. Orthrus was trained using mixed-precision.

All Orthrus models are pre-trained using the Adam optimizer with default hyperparameters for 20,000 steps. Model weights were checkpointed every two thousand steps, and we select the final checkpoint based on validation contrastive loss. We note that the validation loss tended to plateau near the maximum step limit.

The model learning rate was selected from a grid of [1e-3, 1e-4, 1e-5] using validation loss, with a final value of 1e-3 used. We used a linear warm up scheduler with a factor of 0.1 for 2000 steps, and then cosine annealed learning rate for the remaining steps. We found weight decay to be important for constructing a robust latent representation, and a value of 5e-5 and 1e-5 was used for Large and Base respectively. The projection head uses a weight decay value of 1e-4. Gradient clipping was performed, with a norm of five used.

### A.2 Pre-training Augmentation Ablation Experiments

We investigate the Orthrus augmentations that contribute towards effective performance. Here, we perform an ablation study by pre-training Orthrus Base models while withholding one of the utilized augmentations. The Base model is chosen due to computational limitations, and we note that the model may not have sufficient capacity to represent the joint splice and orthology dataset. The results of this analysis are shown in Table A1. We find that augmenting with both orthology and alternative splicing isoforms results in high performance. In addition, the six track representation has higher performance impact for RNA property prediction tasks that are known to be influenced by exon junction density such as RNA half-life prediction [71]. Introduction of masking improves all around performance, which could be due to its prevention of shortcut learning [15, 72].

**Table A1:** Effect of Orthrus pre-training augmentations on RNA property prediction. Ablation results are evaluated using linear probing on embeddings from ablated Orthrus models. Six track input corresponds to using one hot encoded sequence, splicing and codon positions. Masking corresponds to randomly masking 15% of the input sequence.

Splice	Orthology	Six track	Masking	mRNA HL Human R	mRNA HL Mouse R	MRL R	GO MF ROC AUC	Protein Loc. ROC AUC
**✓**	**✓**	**✓**	**✓**	0.675	0.615	0.393	0.845	0.834
**✗**	**✓**	**✓**	**✓**	0.678	0.610	0.392	0.842	0.833
**✓**	**✗**	**✓**	**✓**	0.680	0.615	0.402	0.854	0.834
**✓**	**✓**	**✗**	**✓**	0.531	0.512	0.361	0.833	0.825
**✓**	**✓**	**✓**	**✗**	0.647	0.595	0.332	0.836	0.822
**✗**	**✗**	**✗**	**✗**	0.217	0.214	0.114	0.753	0.792

### A.3 Linear Probe Experimental Details

In this section, we describe the experimental procedure to evaluate linear probing results.

We first performed a 70–15-15 data split on datasets. The data sequences are then embedded by the various self-supervised learning (SSL) models. For Orthrus, we simply take the mean of the embeddings across the seqeunce dimension. For HyenaDNA, we take the mean and max of the embedding sequence dimension, as well as the last hidden state in the output sequence. Other SSL methods could not handle input sequences of more than 500 or 1000 nucleotides. Thus, when input sequences exceeded the allowable context window, each sequence was chunked to the maximum length allowed by a model. We then computed the mean of each chunk embedding across the sequence dimension, and then averaged the mean embedding of each chunk to obtain the final embedding.

After obtaining embedding vectors, we used the scikit-learn implementation of linear models to perform the linear probes of the embeddings. For the downstream regression tasks, we used either used linear regression or ridge regression with the regularization parameter selected by cross validation. The final linear model was selected using the validation split. The gene ontology and protein localization tasks are multilabel classification tasks. For this, we fit scikit-learn’s LogisticRegression model to the labels using a MultiOutputClassifer, which essentially trains a separate linear classifier for each label class. We use the default logistic regression parameters, and set 5000 maximum iterations for the solver. Table A2 shows the performance numbers that are reported in Figure 2.

To avoid information leakage we perform homology splitting within each species. Additional details are described in A.4.

**Table A2:** Linear probing results for self-supervised methods. The embeddings were computed for each method and then linear models were fit using the corresponding labels for each task. Bolded numbers indicate the best performing model. 95% Confidence intervals generated using 10 runs from random initialization.

Model	RNA HL Human	RNA HL Mouse	MRL R	GO MF ROC AUC	Protein Loc. ROC AUC
DNA-BERT2	0.36 ± 7e-3	0.38 ± 6e-3	0.21 ± 9e-3	0.71 ± 2e-3	0.76 ± 1e-3
NT-500m-1000g	0.26 ± 7e-3	0.26 ± 6e-3	0.11 ± 9e-3	0.66 ± 5e-3	0.71 ± 2e-3
NT-500m-human-ref	0.35 ± 4e-3	0.33 ± 7e-3	0.19 ± 5e-3	0.68 ± 3e-3	0.70 ± 1e-3
NT-2.5b-1000g	0.31 ± 5e-3	0.33 ± 4e-3	0.17 ± 7e-3	0.69 ± 2e-3	0.70 ± 1e-3
NT-2.5b-multi-species	0.32 ± 7e-3	0.36 ± 6e-3	0.18 ± 6e-3	0.71 ± 2e-3	0.69 ± 2e-3
Hyena-32K-seqlen	0.42 ± 6e-3	0.44 ± 5e-3	0.28 ± 7e-3	0.74 ± 3e-3	0.78 ± 1e-3
Hyena-160K-seqlen	0.43 ± 7e-3	0.44 ± 6e-3	0.27 ± 5e-3	0.73 ± 2e-3	0.77 ± 1e-3
Hyena-450K-seqlen	0.45 ± 6e-3	0.45 ± 7e-3	0.28 ± 7e-3	0.74 ± 1e-3	0.78 ± 1e-3
RNA-FM	0.40 ± 6e-3	0.38 ± 6e-3	0.19 ± 6e-3	0.74 ± 4e-3	0.78 ± 1e-3

Orthrus Base 4 track	0.52 ± 2e-2	0.54 ± 2e-2	0.38 ± 5e-3	0.84 ± 6e-3	0.83 ± 9e-3
Orthrus Base	0.67 ± 1e-2	0.64 ± 6e-3	0.41 ± 2e-2	0.84 ± 2e-3	0.84 ± 9e-3
Orthrus Large	**0.69** ± 1e-2	**0.65** ± 1e-2	**0.45** ± 2e-2	**0.86** ± 4e-3	**0.84** ± 9e-3

In addition, to further evaluate the quality of the model latent space we visualized the learned embeddings by performing dimensionality reduction using UMAP (Figure A1) [32]. Each point in the figure corresponds to a transcript and is colored depending on the biochemical property of interest. We observe clear separation of transcripts conditioned on the GC % CDS length, number of exons, and transcript length. This ensures that information regarding important biochemical properties is preserved in the model embeddings even after the length pooling operation.

### A.4 Homology Splitting

To perform homology splitting we first acquire paralog information from Ensembl for a species of interest [70]. Ensembl provides pairs paralog information in the form of gene pairs related through duplication events. However, to perform homology splitting between genes we want to make sure that paralog transitivity is taken into account when dividing training samples between train, validation, and test splits. For example given three genes $g_{1}$, $g_{2}$, $g_{3}$ if $g_{1}$, $g_{2}$ are annotated as paralogs and $g_{2}$, $g_{3}$ are annotated as well we want to ensure that $g_{1}$, $g_{3}$ are in the same split. Thus, we first transform the pairwise relationships into a graph structure in the process pruning low confidence paralog relationships. We enforce a similarity threshold of 35% which empirically demonstrated highly connected groups of paralogs. The algorithm for grouping is described in [27].

Following construction of the homology graph, during train-val-test split samples associated with genes which are connected in the homology graph are indexed into the same split. This avoids information leakage due to homology relationships within a given species.

### A.5 Fine-tuning Experimental Details

We fine-tune Orthrus by first initializing most of the model with weights from pre-training, the penultimate two layers with random initialization, and the final layer with zero initialization. We don’t apply any weight decay to weights that were initialized from pre-training while the final three layers have an l2 weight decay term of 1e-5. We fine-tune on downstream tasks using the Adam optimizer with a learning rate of 0.01. We apply exponential learning rate decay with a factor of 0.95. The models are trained with a single Nvidia T4 GPU in a mixed precision setting.

HyenaDNA models initialized with fine-tuning head. We perform a small learning rate hyperparameter grid search around the suggested hyperparameters of 6e-4. The suggested AdamW optimizer is used. Models were trained for a maximum of 100 epochs on Nvidia T4 GPUs with a batch size of 28 for the HyenaDNA-tiny and a batch size of 8 for HyenaDNA-small. Models were stopped early based on validation loss using an epoch patience of three. After selecting learning rate using the validation split, the runs were repeated using different random initializations to generate confidence intervals.

**Algorithm 1:** Homology Group Assignment

**Input:** Set of genes G, Set of paralogous relationships P, Similarity threshold s
**Output:** Homology map assigning genes to groups
**Initialize:**
group_counter←0
homology_map←{}
**Filter:**
P←{(g1,g2)∈P∣similarity(g1,g2)>s}
**for each** (g1,g2) **in** P **do**
**if** g1∉homology_map **and** *g2∉homology_map* **then**
homology_map[g1]←group_counter
homology_map[g2]←group_counter
group_counter←group_counter+1
**else if** g1∈homology_map **and** g2∉homology_map **then**
homology_map[g2]←homology_map[g1]
**else if** g2∈homology_map **and** g1∉homology_map **then**
homology_map[g1]←homology_map[g2]
**else if** homology_map[g1]≠homology_map[g2] **then**
old_group←homology_map[g2]
new_group←homology_map[g1]
**for each** gene **in** homology_map **do**
**if** homology_map[gene] ==old_group **then**
homology_map[gene]←new_group
**end if**
**end for**
**end if**
**end for**
**Return** homology_map

**Table A3:** Pearson correlations (R) and ROC AUC of full model fine-tuning on mRNA half-life (HL), mean ribosome load (MRL), gene ontology molecular function (GO MF) classification, and protein localization. Best models are shown in bold. Performance values were averaged over 3 random seeds. Training validation and test were split based on sequence homology to prevent data leakage.

Models	mRNA HL Human R	mRNA HL Mouse R	MRL R	GO MF ROC AUC	Protein Loc. ROC AUC	Number of Parameters	Number of Tracks
Mamba Base Supervised	0.65 ± 2e-2	0.62 ± 1e-2	0.46 ± 2e-2	0.8 ± 2e-2	0.82 ± 3e-4	1.3m	6
Dilated CNN Resnet	0.68 ± 9e-3	0.66 ± 4e-3	0.13 ± 1e-1	0.81 ± 1e-2	0.8 ± 6e-3	0.83m	6
Saluki	0.68 ± 2e-2	0.63 ± 2e-2	0.41 ± 8e-2	0.79 ± 2e-2	0.82 ± 2e-3	0.15 m	6
HyenaDNA Small	0.48 ± 5e-2	0.48 ± 2e-2	0.44 ± 5e-2	0.78 ± 2e-2	0.81 ± 7e-3	3.3m	4
Orthrus Base	**0.74** ± 9e-4	**0.71** ± 4e-3	**0.52** ± 2e-2	**0.84** ± 2e-3	**0.83** ± 6e-5	1.3 m	6
